# To Each His Own: DEHP Yields Species-Specific Metabolic Phenotypes

**DOI:** 10.1289/ehp.118-a81a

**Published:** 2010-02

**Authors:** Julia R. Barrett

**Affiliations:** **Julia R. Barrett**, MS, ELS, a Madison, Wisconsin–based science writer and editor, has written for *EHP* since 1996. She is a member of the National Association of Science Writers and the Board of Editors in the Life Sciences

Endocrine disruptors have been shown to disturb the balance between energy expenditure and storage in cellular models, a balance that is critical for proper metabolic functioning. Peroxisome proliferator–activated receptors (PPARs), potential molecular targets of endocrine disruptors in several tissues and organs, hold a key position as lipid sensors that direct metabolic gene expression. A new mouse study illustrates activation of a specific PPAR isotype with exposure to the endocrine disruptor diethylhexyl phthalate (DEHP) and provides evidence that the potential influence of DEHP exposure on diet-induced obesity may vary between species **[*EHP*** **118:234–241; Feige et al.]**.

DEHP is a widely used industrial plasticizer that can leach from diverse consumer products including food packaging and medical devices such as plastic tubing and bags. When ingested, DEHP is converted to monoethylhexyl phthalate (MEHP), which is readily absorbed. Previous *in vitro* research has demonstrated that MEHP can activate all three PPAR isotypes (PPARα, PPARβ, and PPARγ). The result *in vivo* can be opposing effects depending on which isotype is activated: induction of adipogenesis (PPARγ) or fatty acid oxidation (PPARα, PPARβ).

To determine the physical and biochemical effects of DEHP exposure, weanling mice were fed regular diets, with treatment groups receiving either 100 mg DEHP/kg/day (low dose) or 1,000 mg DEHP/kg/day (high dose) in the chow. Food intake and physical activity did not differ between groups, and lean body mass was not affected. However, in DEHP-treated mice fat reserves were reduced, and blood tests indicated increased hepatic fatty acid oxidation. As a result, mice in the high-dose group gained 15% less weight than low-dose and control mice over the 10-week treatment period.

In a separate experiment, adult mice were fed a high-fat diet for 13 weeks. Fat mass increased from a baseline 8–10% of body mass to 30% in untreated mice but remained unchanged in mice receiving 500 mg DEHP/kg/day. Further experiments investigating the pattern of PPAR target gene expression and using mice lacking either PPARα or PPARβ revealed that DEHP effects were mediated through PPARα in the liver.

Finally, to make the model more applicable to humans, mice genetically engineered to carry human PPARα were exposed to DEHP. Interestingly, MEHP did not protect these mice from diet-related obesity as it did in wild-type mice; in fact, MEHP led to these mice being even more obese than controls. If this relationship holds true in humans, exposure to certain endocrine disruptors could potentially contribute to obesity by promoting fat accumulation.

Conclusions drawn from this study include the identification of hepatic PPARα as a key site for DEHP-associated disruption. The doses applied in this study are 2–3 orders of magnitude higher than estimated typical human exposures when normalized to body mass. However, the observation of subtle, species-specific differences in metabolic response to DEHP point to an important factor that should be considered as the biological effects of DEHP on human health are further explored.

